# Evaluation of brewers’ spent grain as a novel media for yeast growth

**DOI:** 10.1186/s13568-017-0414-1

**Published:** 2017-06-05

**Authors:** Sachindra T. Cooray, Jaslyn J. L. Lee, Wei Ning Chen

**Affiliations:** 1 Interdisciplinary Graduate School, Nanyang Technological University, 50 Nanyang Avenue, Singapore, 639798 Singapore; 2 Advanced Environmental Biotechnology Centre, Nanyang Environment & Water Research Institute, Nanyang Technological University, 1 CleanTech Loop, Singapore, 637141 Singapore; 3 School of Chemical and Biomedical Engineering, Nanyang Technological University, 62 Nanyang Drive, Singapore, 637459 Singapore

**Keywords:** Brewers’ spent grain, Growth media, *Rhodosporidium toruloides*

## Abstract

Brewers’ spent grain (BSG) is a by-product generated from the beer manufacturing industry, which is extremely rich in protein and fiber. Here we use low cost BSG as the raw material for the production of a novel growth media, through a bioconversion process utilizing a *food grade fungi* to hydrolyze BSG. The novel fermentation media was tested on the yeast *Rhodosporidium toruloides*, a natural yeast producing carotenoid. The yeast growth was analysed using the growth curve and the production of intracellular fatty acids and carotenoids. Untargeted GCMS based metabolomics was used to analyse the constituents of the different growth media, followed by multivariate data analysis. Growth media prepared using fermented BSG was found to be able to support the growth in *R. toruloides* (21.4 mg/ml) in comparable levels to YPD media (24.7 mg/ml). Therefore, the fermented BSG media was able to fulfill the requirement as a nitrogen source for *R. toruloides* growth. This media was able to sustain normal metabolomics activity in yeast, as indicated by the level of fatty acid and carotenoid production. This can be explained by the fact that, in the fermented BSG media metabolites and amino acids were found to be higher than in the unfermented media, and close to the levels in YPD media. Taken together, our study provided evidence of a growth media for yeast using BSG. This should have potential in replacing components in the current yeast culture media in a sustainable and cost effective manner.

## Introduction

BSG (Brewers’ Spent Grain) is the protein and fiber rich residue, accounting to 20 and 70% of dry weight respectively, produced during the process of beer manufacturing. The availability of spent grain is increasing with the increase in the beer production levels. Alternative uses for BSG are highly sought-after due to the increasing cost of disposal to be incurred.

Malted barley, the most common ingredient used to produce beer, is made by germinating barley. Afterwards, the grains are dried and crushed to remove the germ. This mixture is then treated in hot water, at 70–74 °C where the grain starch is converted to fermentable sugars (mashing step). The filtered sugar-rich liquid also known as the wart is fermented to produce beer, and the rejected retentate is known as the brewers’ spent grain. World beer production was reported to be 193 billion liters in 2014 and the globally leading countries in beer production are China, United States and Brazil (FAO 2017). Furthermore, for every 100 L of beer produced 20 kg of BSG is generated during processing (Mussatto et al. 2006). Thus, BSG is generated in massive quantities throughout the world. BSG accounts for approximately 85% of the by-products generated in the brewery industry (Mussatto et al. 2006) and for 30–60% of BOD (biological oxygen demand) and suspended solids generated by a typical brewery (Aliyu and Bala 2010).

BSG is a lignocellulosic material rich in protein and fiber (cellulose, arabinoxylan and lignin) accounting for approximately 20 and 70% respectively. BSG is reported to contain vitamins (biotin, folic acid, niacin, choline, riboflavin, thiamine, pantothenic acid and pyridoxine) and minerals (calcium, cobalt, copper, iron, magnesium, manganese, phosphorus, potassium, selenium, sodium and sulphur). Both essential (including lysine, histidine, methionine, phenylalanine and tryptophan) and non-essential (including alanine, serine, glycine and proline) amino acids are also reported to be present in BSG (Mussatto et al. 2006). BSG is rich in phenolic compounds (principally ferulic acid and *p*-coumaric acid) along with oligo-saccharides and polysaccharides. Evidence of dietary phenolic compounds to exhibit anti-carcinogenic, anti-inflammatory and antioxidant activities has been found recently. Similar to other lignocellulose material BSG is reported to contain various valuable materials within. One of the main reasons hindering the possessing of those is due to the recalcitrance of plant cell walls. Therefore, physical, chemical or biological conversion needs to be performed on the BSG.

Being available throughout the year at low or no cost at all makes spent grain a favourable raw material for any potential application. The majority of the produced BSG is currently disposed in landfills or used as feed for animals such as cattle, pig and chicken (Mussatto et al. 2006). Other alternative uses include energy production, through direct combustion or biogas (Čater et al. 2015), bioethanol production (Liguori et al. 2015), for charcoal (Zhang et al. 2015), activated carbon production (Mussatto et al. 2010; Vanreppelen et al. 2014), paper manufacturing (Russ et al. 2005; Sousa et al. 2007), as a brick component (Russ et al. 2005), to grow mushroom on (Beharry 2015) and composting for agriculture to be used as fertilizer (Beharry 2015).

Yeast extract-peptone (YP) is the conventional growth media used for fungi species, in producing a range of bio products, such as succinic acid (Vlysidis et al. 2009), microbial oil (Saenge et al. 2011) and valuable metabolic products (Chatzifragkou et al. 2011). Yeast extract is made through autolysis (European Association for Specialty Yeast Products 2016), where grown yeast is heated to 45–55 °C. During this stage, yeasts proteins are denatured by endogenous digestive enzymes and allowed to mix with the aqueous solution. Finally the solution is purified and converted to a paste or concentrated liquid. Yeast extract is enriched with proteins, amino acids, vitamins (B1, B2, B6, niacin, folic acid, pantothenic acid and biotin) and minerals (potassium, sodium, calcium, magnesium, iron and zinc) from the original yeast. Peptones are a widely used nitrogen source for microbial media. Most peptones are made by incubating milk or meat with trypsin, pepsin or other proteolytic enzymes, to digest proteins into amino acids, peptides and polypeptides, but sometimes also by cooking milk or meat in acid. There exist a variety of peptones, such as plant-based peptones (made using potatoes, soybeans and wheat), meat peptones (made from a porcine, bovine or poultry origin), casein peptone (made from animal sources) and gelatin peptone (made by boiling collagen, isolated from animal skin, bones and tissues, and subsequent pancreatic digestion) (Gray et al. 2008). However, it is expensive to use YPD media in large quantities industrially. Thus, development of a novel cost effective media to support yeast growth will be favourable for many applications in industrial scale.

The objective of this study was to develop a novel use for the BSG to be used as a growth media for the yeast strain, *Rhodosporidium toruloides*. Possibility of introducing an inexpensive substitute for YPD media from BSG would allow reducing the cost related to using expensive growth media. Alternatively such a novel media would enable to decrease the competition for food sources and natural resources, while establishing alternative uses for a food waste material such as BSG.

## Materials and methods

### Brewers’ spent grain

BSG was kindly provided by Asia Pacific Breweries (Singapore) Pte, Ltd. and was stored in airtight plastic containers at −80 °C till used.

### Strains

Yeast strain *R. toruloides* (CBS 5490, Central Bureau voor Schimmelcultures, Utrecht, The Netherlands) were maintained on agar plates containing (per liter) 20 g of agar (Sigma, St. Louis MO, USA), 10 g of yeast extract (Biobasic Canada Inc.), 20 g of peptone (Biobasic Canada Inc.) and 20 g of d-dextrose (Sigma, St. Louis MO, USA).

### Medium and culture conditions

All growth media were prepared with deionized water. The biofermentation and the extraction of nutrient for the preparation of BSG growth media were performed according to a method proposed (Kirana et al. 2012) with minor modification. The unfermented BSG media was prepared using the same method for the nutrient extraction. The extracts were centrifuged (14,500 rpm, 20 min, 4 °C), filtered through a 0.45 μm filter and autoclaved (121 °C, 20 min). These solutions were used as the fermented BSG media and the unfermented BSG media to grow *R. toruloides* in. YPD fermentation media was prepared with 20 g of peptone, 10 g of yeast extract and 20 g of dextrose (per liter).

*Rhodosporidium toruloides* was initially grown on YPD agar plates (2% glucose), made of 10 g of yeast extract, 20 g of peptone, 20 g of agar and 20 g of dextrose dissolved in 1000 ml of ultrapure water. Overnight culture of *R. toruloides* grown in YPD fermentation media (2% w/w glucose) was used to inoculate 50 ml of the fermentation media with an optical density (OD_600_) of 0.2. Fermentation was carried out at 30 °C in 250 ml Erlenmeyer flasks incubated for 5 days shaken at 200 rpm. Three biological replicates were carried out during the experiments.

### Growth analysis

The growth in the yeast cultures was found out using the optical density (OD_600_) of the culture media. Samples were collected every 24 h for 5 days and OD_600_ was measured using NanoDrop 2000 UV–Vis spectrophotometer (Thermo Scientific, Waltham, MA, USA). The OD_600_ values were correlated to dry weight to construct the growth curve for the yeast. Samples with varying concentrations of cells were centrifuged (10,000*g*, 5 min), washed twice with ultrapure water and analysed using the infrared moisture analyser (Sartorius MA37) to find the dry cell weight. This data was used in constructing a calibration curve for OD_600_ values and yeast cell dry weight.

### Carotenoid measurement

1 ml of the culture was used to measure the intracellular carotenoid production by *R. toruloides*. The samples were centrifuged for 10 min at 10,000 rpm, 4 °C and washed with Milli-Q water. The cell palettes were broken down using glass beads in the Fast Prep Grinder (MP Biomedicals, Solon, OH, USA). Carotenoids were extracted with 1 ml of acetone until the cell pallets were colourless.

Agilent 1100 high-performance liquid chromatography (HPLC) equipped with a photodiode array detector was used to identify and quantitate the carotenoids extracted. Carotenoids, torularhodin, torulene and β-carotene, were identified using standards from CaroteNature (Ostermundigen, Switzerland). Li-Chrospher 100RP-18 column (250 mm × 4.6 mm id, 5 μm) and a guard column (4 mm × 4 mm id) of the same material (Merck, Rahway, NJ, USA) were used for the separation. Acetone and Milli-Q water were used as the mobile phases with a gradient from 70 to 100% (acetone in Milli-Q water) at a 0.5 ml/min flowrate. Detection was performed at 450 nm, and the UV–Vis absorption spectra were recorded online using the photodiode array detection system (Lee et al. 2014).

### Fatty acid measurement

Lipids were extracted from the yeast cells employing the chloroform–methanol 2:1 method adopted by Chen and Chen (2014). 1 ml of the culture medium was extracted from the fermentation flask. The cell pallets were washed three times using Milli-Q water. These washed cells were centrifuged for 10 min at 10,000 rpm, 4 °C and separated. The cell pallets were resuspended in 1 ml of 0.9% NaCl and acidified with 200 μl of acetic acid. 10 μl of 10 mg/ml of heptadecanoic acid (dissolved in ethanol) (Sigma, St. Louis MO, USA) was added as the internal standard (IS) to account for the fatty acid loss during processing. Around 300 μl of glass beads were added and the cells were disrupted in the FastPrep^®^-24 instrument for 30 s for four times.

3 ml of a chloroform–methanol (2:1) mixture was added to the samples, inverted several times, vortexed vigorously and centrifuged (10,000*g*, 10 min, 4 °C). The bottom chloroform layer was transferred to another tube. This step was repeated once again. The collected chloroform was dried overnight to dryness on a heatblock at 30 °C.

Fatty acid derivatization was performed according to a method proposed by Horak et al. (2009). The dried lipid residue was redissolved in 500 μl 10% BF3-methanol and incubated in a sealed screw cap tube in a heatblock at 95 °C for 20 min. Then the tubes were cooled down to near room temperature. 300 μl of saturated NaCl in water and 300 μl of n-hexane were added. Samples were centrifuged (14,000 rpm, 10 min) at room temperature. The upper hexane layer containing the extracted Fatty acid methyl esters (FAMEs) were transferred to glass vials for GCMS analysis. FAME mix C8-C24 (Sigma, St. Louis MO, USA) was used as the standard for quantitation.

Chromatography was performed using Agilent Technologies 7890A GC-5975C inert MS system. 1 μl samples were injected into the HP-5MS capillary column by splitless mode using an auto-injector. Helium was used as a carrier gas at a flow rate of 1.1 ml/min. The inlets and MS source temperatures were maintained at 250 and 230 °C respectively. The oven temperature was maintained at 80 °C for 1 min and ramped to 250 °C at a rate of 7 °C/min, then held at 250 °C for 10 min. Data were acquired in full scan from 35 to 600 m/z.

### Media metabolite analysis

1.5 ml of the original sample media was spiked with 10 µl internal standard (IS, ribitol, 2 mg/ml dissolved in water) and freeze dried. The lyophilised samples were derivatized for GCMS analysis according to Wang et al. (2010). Methoximation was performed by dissolving the samples in 50 µl of methoxyamine hydrochloride (20 mg/ml in pyridine) (Sigma, St. Louis MO, USA) to protect the carbonyls and incubating at 37 °C for 60 min. Afterwards, silylation was carried out by adding 100 µl of *N*-methyl-*N*-(trimethylsilyl)-trifluoroacetamide (MSTFA) with 1% trimethylchlorosilane (TMCS) (Sigma, St. Louis MO, USA) to each sample and incubating at 70 °C for 30 min. Subsequently the samples were shaken for 60 min at room temperature and then analysed in GCMS. All samples were analysed within 24 h in a random order.

Metabolites were analysed using Agilent 7890A GC-5975C inert MSD (with Triple Axis Detector) system (Agilent Technologies, CA, USA) equipped with a HP-5MS, 5% Phenyl-Methyl-Silox capillary column (30 m × 250 μm × 0.25 μm Agilent J&W Scientific, Folsom, CA, USA). 1 μl samples were injected to the system by the auto-sampler in split less mode. The solvent cut off was set to 5 min. Helium was used as a carrier gas at a flow rate of 1.1 ml/min. The inlet and ion source temperatures were maintained at 250 and 230 °C. The oven temperature was maintained at 75 °C for 4 min and increased at 4 °C/min to 280 °C and remained for 2 min. Data was acquired in full scan from 35 to 600 *m/z*.

Chromatographic peak deconvolution and identification was processed using Agilent MassHunter Qualitative Analysis software (B.06.00). Multivariate data analysis and statistical analysis were performed using Mass Profiler Professional (B.02.01) software. The list of compounds extracted was subjected to alignment, normalization (according to the IS, ribitol) and filtering. Afterwards, principal component analysis (PCA) was performed on the obtained data to eliminate any outliers present in the data. Clustering heatmap was created using K-Means algorithm and Euclidean distance calculation. Statistical analysis of the data were performed by one-way ANOVA followed by the post hoc Tukey’s Honest Significant Difference (HSD) to determine significant metabolic changes; p < 0.05 was considered significant. The multiple testing was corrected by Benjamin Hochberg false discovery rate (FDR). Accurate masses of the compounds were searched against NIST mass spectral library with similarity above 75% for feature identification.

## Results

### Growth of yeast in unfermented BSG media

**Fig. 1:**
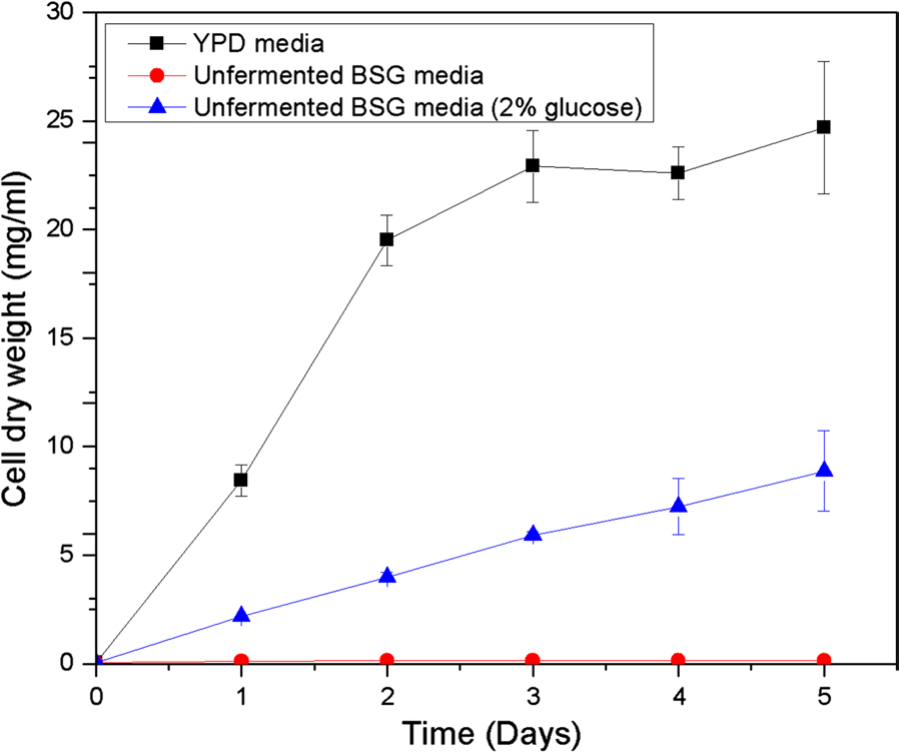
Growth curves for *R. toruloides* when grown in YPD, unfermented BSG and unfermented BSG (​2% w/w glucose) media represented as cell dry weight (mg/ml) vs. time (days)

Initially, nutrients were extracted from original BSG (unfermented) to produce a growth media. *R. toruloides* was cultured in the media under controlled parameters and monitored daily over a period of 5 days using the optical density of the culture. The hypothesis was that the nutrient extract was able to provide all growth nutrients for yeast, being the sole carbon source and sole the nitrogen source. No growth was observed in yeast when compared with the growth in conventional YPD media (2% w/w glucose). Afterwards, another trial was carried out, to use the original BSG nutrient extract only as a nitrogen source, where a carbon source (2% w/w glucose) was added externally. However, the observation was that the growth was remarkably low relative to the growth in YPD. Therefore, media from original BSG was unsatisfactory to be used as growth media. Figure 1 depicts the growth observed in *R. toruloides* during the two trials.

### Growth of yeast in fermented BSG media

**Fig. 2:**
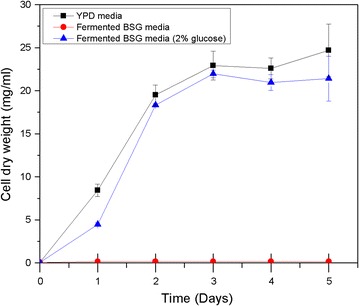
Growth curves for *R. toruloides* when grown in YPD, Fermented BSG and Fermented BSG (2% w/w glucose) media represented as cell dry weight (mg/ml) vs. time (days)

Afterwards, fermented BSG was used to produce the growth media. Initially, the fermented BSG media was used as the sole nutrient source (both carbon and nitrogen) to grow *R. toruloides*. But, the media was unsuccessful as no growth was observed. Similar to the previous trial with unfermented BSG, then an external carbon source (2% w/w glucose) was added and the experiment was repeated. It was seen that the growth was improved in *R. toruloides*. Moreover, the yeast strain was able to grow at a similar level as in YPD media according to the observations. The growth curve for *R. toruloides* in fermented BSG media is illustrated in Fig. 2.

### Fatty acid production

*Rhodosporidium toruloides*, being an oleaginous microorganism is reported to accumulate lipids. When the yeast cells were analyzed it was observed that methyl esters of oleic acid (C18.1) and linoleic acid (C18.2) were present. The total amount of fatty acids produced from *R. toruloides* in varied media is summarized as shown in Table 1 (indicated as mean ± standard deviation).

**Table 1:** Production of fatty acids by *R. toruloides* in the three different culture media at the end of the fermentation, indicated as mean ± SD

Culture media	Fatty acid content (%) (g lipid/g biomass)	Fatty acid yield (g lipid/l)
YPD media	44.36 ± 3.29	8.69 ± 0.23
Fermented BSG media	35.31 ± 2.64	7.03 ± 0.32
Unfermented BSG media	22.18 ± 1.19	1.69 ± 0.17

According to the results, YPD media showed the highest level of fatty acid production (8.69 g/l) by yeast, but the yeast grown in fermented BSG media were able to produce fatty acids in levels (7.032 g/l) comparable to the YPD media. However, *R. toruloides* produced significantly low amount of fatty acids (1.69 g/l) in the unfermented BSG media. Lipid content (% g/g biomass) variation showed a similar descending pattern by *R. toruloides* grown in YPD, fermented BSG and unfermented BSG media respectively.

### Carotenoids production

**Fig. 3:**
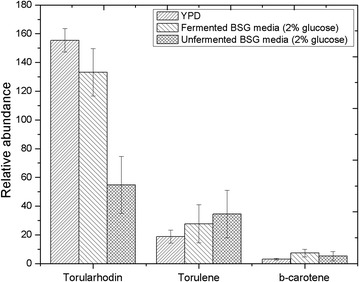
Relative production of carotenoids; torularhodin, torulene and β-carotene, by *R. toruloides* when grown in YPD, fermented BSG (2% w/w glucose) and unfermented BSG (2% w/w glucose) media at the end of 5-days fermentation

Carotenoids are an important type of metabolite produced by *R. toruloides*. The HPLC analysis confirmed the production of three types of carotenoids; torularhodin, torulene and β-carotene. A qualitative analysis of these three-types suggested similar level of carotenoids production even if the nitrogen source of the fermentation media was changed. Yeasts in YPD media was able to produce the highest amount of torularhodin closely followed by yeasts grown in fermented BSG media. An opposite pattern was observed in torulene, where *R. toruloides* grown in unfermented BSG produced the highest quantity. The level of β-carotene production was quite close within all three test subjects. The results obtained are illustrated in Fig. 3.

### Metabolite profiling of growth media

**Fig. 4:**
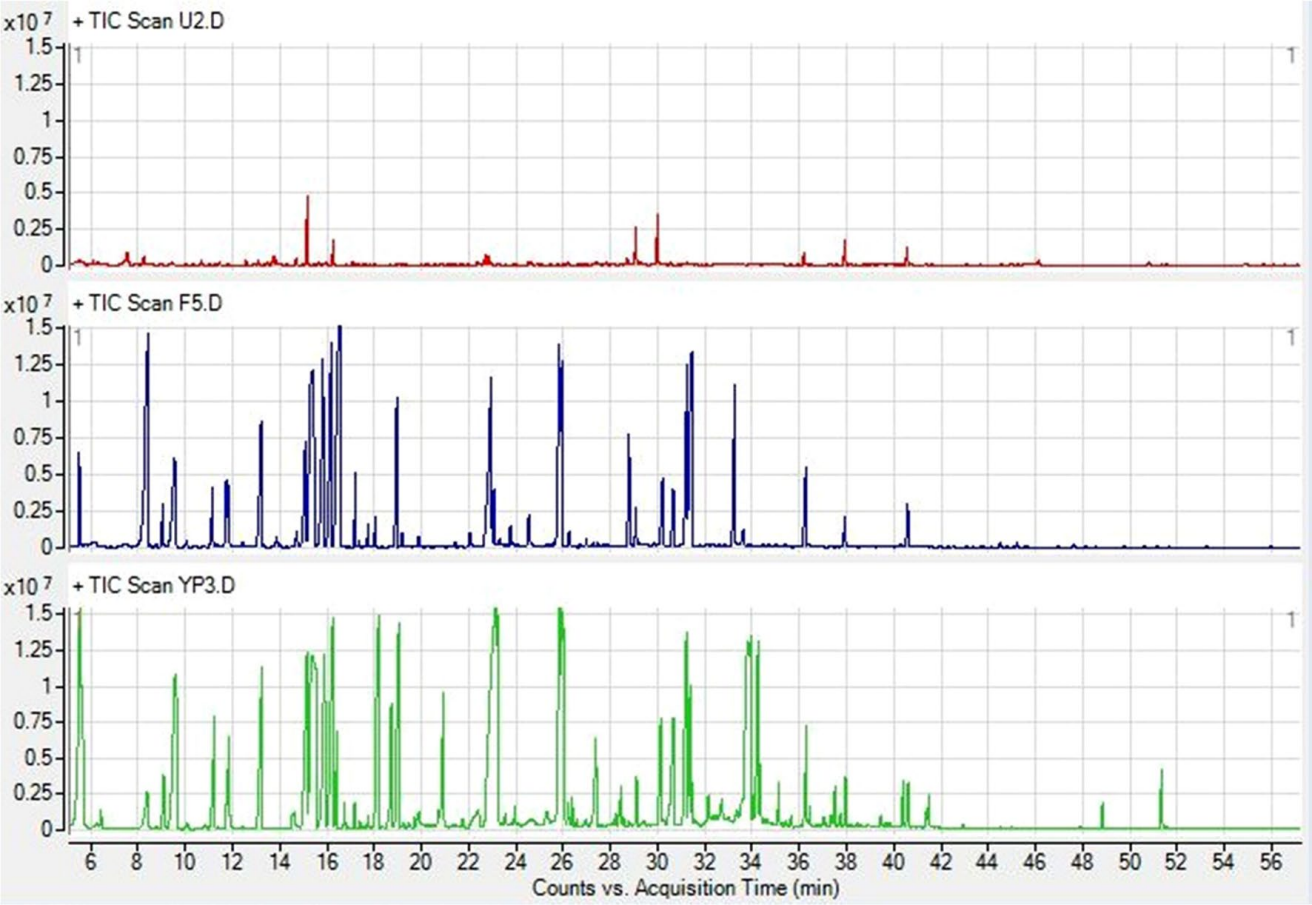
GCMS chromatograms observed of unfermented BSG media (*top*), fermented BSG media (*middle*) and YP media (*bottom*) when examined to detect metabolites present within

Metabolome of the YP media, fermented BSG media and unfermented BSG media were examined to gain insight into their variation. The studies were established using GCMS results and multivariate data analysis. Figure 4 illustrates the spectra of the three-different media overlaid together. Even if the visual inspections show that there are changes among the media a reliable method, multivariate analysis, was used to accurately describe the variations.

Principal component analysis (PCA) is a commonly used method to reduce the raw data dimensionality into few principal components describing the maximum possible variation in the data.

**Fig. 5:**
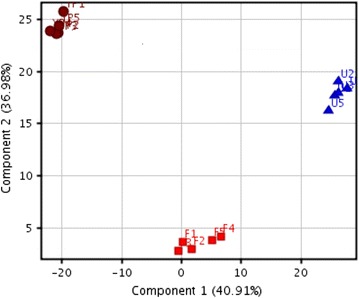
PCA plot for extracted metabolites from the different media and investigated using GCMS. Component 1 vs. Component 2 of YPD media (*brown circle*), Fermented BSG media (*red square*) and Unfermented BSG media (*blue triangle*)

**Fig. 6:**
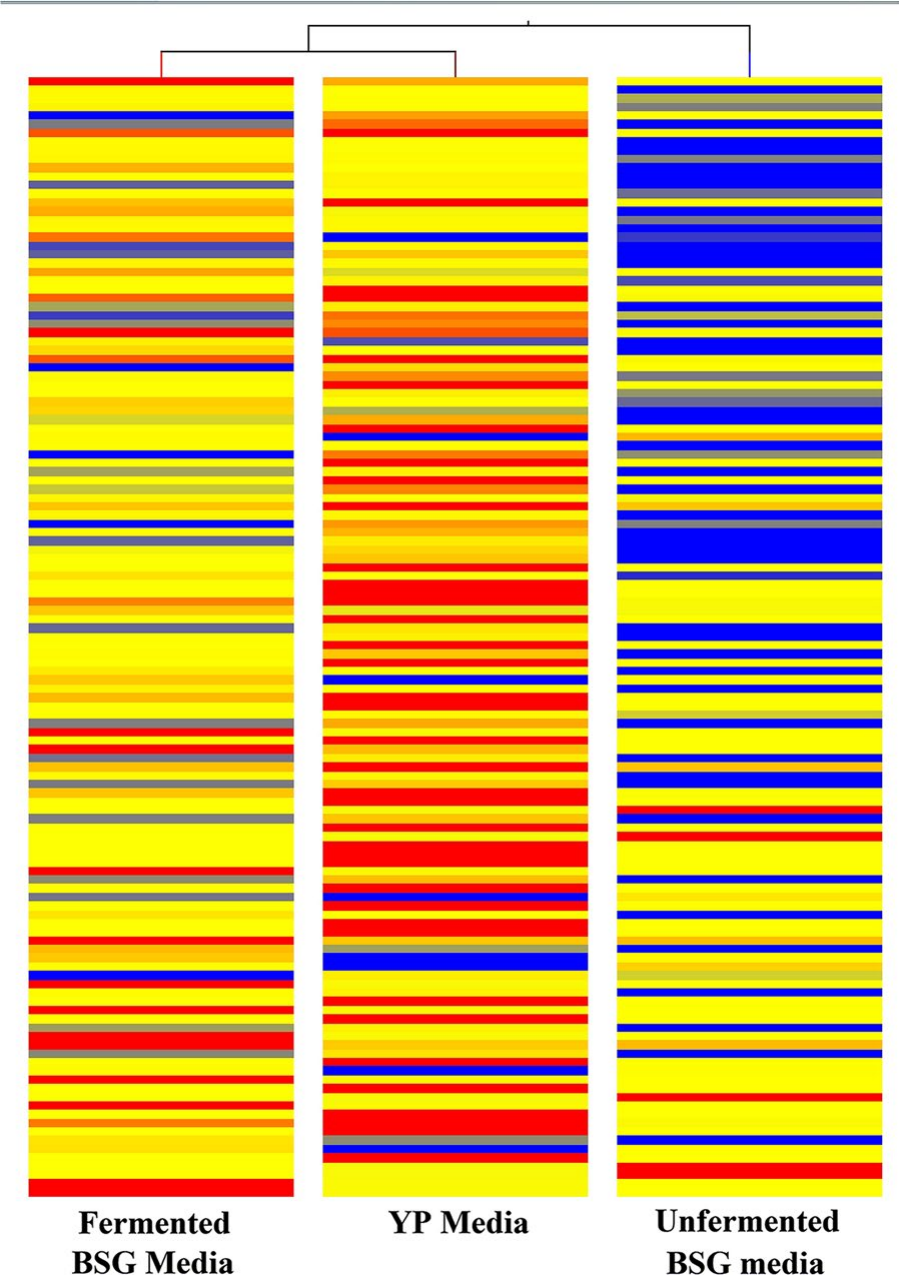
Heatmap correlation of metabolites extracted by GSMS spectra in YP media, unfermented BSG media and fermented BSG media. Metabolites shaded in *red* are up regulated while those in *blue* are down regulated in terms of abundance

PCA scores plot (Fig. 5) clearly depicted that the three media considered in this study inherited varying properties among them. All significantly differentiating metabolites determined were visualised using hierarchical cluster analysis (HCA) as shown in Fig. 6. The HCA diagram validated the increased metabolite concentrations in the fermented BSG media after the biofermentation process and its relative proximity to YP media.

Out of 129 metabolites aligned from the analysis only 38 were identified through the library search. The identified differential metabolites found in fermented BSG media, unfermented BSG media and YP media are represented in Table 2.

**Table 2:** List of identified metabolites extracted from the growth media with their relative abundances

Compound name	Fermented BSG media	Unfermented BSG media	YP media
2-Butenedioic acid	47,556		7495
2-Hydroxyisocaproic acid	428,585	703,628	
Gluconic acid	42,443,796	65	5
Adenine	5		4,528,553
Alanine	92,608,400	47,046	79,485,704
Asparagine	232,784	950	27,334,972
Butanedioic acid	3,847,280		30
Glycine	267,426	466,725	448,586
l-5-Oxoproline	29,703,624	363	80,928
l-Cysteine	739,977		
l-Glutamic acid	82,777,448		70,706,632
l-Leucine	6023		44,094,996
l-Lysine	646		656,699
l-Ornithine	6,495,508	2539	77,047,232
l-Threonine	43,247,852	96	85,696,200
l-Tyrosine			65,736
l-Valine	753,659		608,750
Maltose		47	4,080,956
Phenylalanine	4,407,764		58,384,488
Phosphonic acid	2996		20,095,596
Phosphoric acid	69	703,758	30,840,496
Propylene glycol	95,308	293,640	
Putrescine	32,753,962	6894	380,827
Serine	691		92,337,480
Stearic acid	9,795,037	4,565,880	775,570
Uracil	9,385,660	2289	4,053,908

## Discussion

This study investigated the suitability of a fermentation media produced by BSG to sustain the growth of the yeast *R. toruloides*. The media produced using the unfermented BSG was not able to fulfill both the carbon and nitrogen requirements for the growth of yeast.

On the other hand, fermented BSG growth media showed exceptionally better growth of *R. toruloides* compared to unfermented BSG growth media, and showed similar growth patterns when compared with YP media. However, according to the growth curve (Fig. 2) fermented BSG media was only able to fulfill the nitrogen requirement for yeast growth and had to be supplied with an external carbon source (2% w/w glucose). Therefore, the prior bioconversion process was an essential step to release the nutrients from BSG. Thus, the fermentation on the BSG is presumed to hydrolyze proteins into peptides and amino acids due to the production of proteases by the fungi growth.

Hence, it is clear that the bioconversion has helped to break down the BSG proteins and increase the nitrogen content. This should be due to proteases secreted by the fungi used to treat the BSG during solid-state fermentation. Fungi such as *Aspergillus oryzae* are reported to produce high amounts of enzymes such as, proteases that hydrolyze proteins. These fungi are also known to produce lipase, phytase, xylanase, β-galactosidase, cellulase and amylolytic enzymes. In addition to the fermentation, the autolysis also helped in order to further break down proteins in the spent grain. Furthermore, the limitation in oxygen in the media during the extraction process helped the autolysis of the fungi encouraging regeneration of microbial nutrient from biomass (Koutinas et al. 2005).

### Fatty acid production

The total fatty acid production by *R. toruloides* in the fermented BSG (7.03 mg/ml) was close to the levels produced by the yeasts grown in YPD (8.69 mg/ml), unlike the low levels produced by the yeasts grown in unfermented BSG media (1.69 mg/ml). The yeasts grown in fermented BSG media also demonstrated an improvement in fatty acid content (35.31% g/g cell dry mass) when compared to those in unfermented BSG media (22.18% g/g cell dry mass).

It is known that oleaginous yeasts begin accumulating lipids when the nitrogen source is exhausted. Therefore, there exists an inverse correlation between the cell mass production, which depends on the initial nitrogen content, and the fatty acid accumulation by the cells, depending on the C/N ratio of the media. In oleaginous yeasts lipid accumulation could occur via 2-different pathways. (1) de novo synthesis, acetyl-CoA and malonyl-CoA building blocks are used for the lipid synthesis, and (2) ex novo lipid accumulation pathway, in which fatty-acids, oils and tricarboxylic acids from culture media are involved. Huang reports that the organic nitrogen source used in fermentation affects the lipid production (Huang et al. 2010). Moreover, organic nitrogen sources tend to increase lipid production while inorganic nitrogen sources favour cell growth (Evans and Ratledge 1984; Fakas et al. 2008a). When sugars are used as the carbon source oleaginous yeasts are reported to produce unsaturated fatty acids such as, oleic acid (C18.1) and linoleic acid (C18.2), which is more valuable in terms of biological function and economical value (Anschau 2017). It is being reported that the main requirement for high lipid production rate is for the medium to contain excess of the carbon source while under nutrient conditions for the nitrogen source, maintaining a high C/N ratio (Fakas et al. 2008b). Trace metal ions such as Mn^2+^, Mg^2+^, Cu^2+^, Zn^2+^ and Ca^2+^, are reported to influence the biomass and lipid accumulation (Li et al. 2006). This observation in the study stated that the fermented BSG was able to provide the adequate nutrient requirement for normal metabolomics activities in *R. toruloides*.

### Carotenoid production

In the carotenoid synthesis pathway Lycopene is the branch point of β-carotene and torulene/torularhodin pathways, and torularhodin is produced by further oxidation of torulene. According to Fig. 3, the YP media and fermented BSG media must be promoting further oxidation of torulene, unlike the unfermented BSG media.

Carotenoids being a secondary metabolite, in most yeasts initiate production during the late logarithmic phase and continue till the end of stationary phase. Media with a high C/N ratio tends to promote lipid production rather than carotenoids. However, as growth progress the C/N ratio changes promoting production of secondary metabolite such as carotenoids (Somashekar and Joseph 2000). Higher quantities of carotenoids are produced when inorganic nitrogen salts are used. A study reported the highest level of β-carotene production when NH_4_NO_3_ was used as the nitrogen source and highest level of torulene production when peptone was used (El-Banna et al. 2012). The supplementation of the growth media with amino acids (alanine and threonine) tends to increase the carotenoid production, which could be as a result of amino acids influencing the pigment formation (Voaides and Dima 2012).

**Fig. 7:**
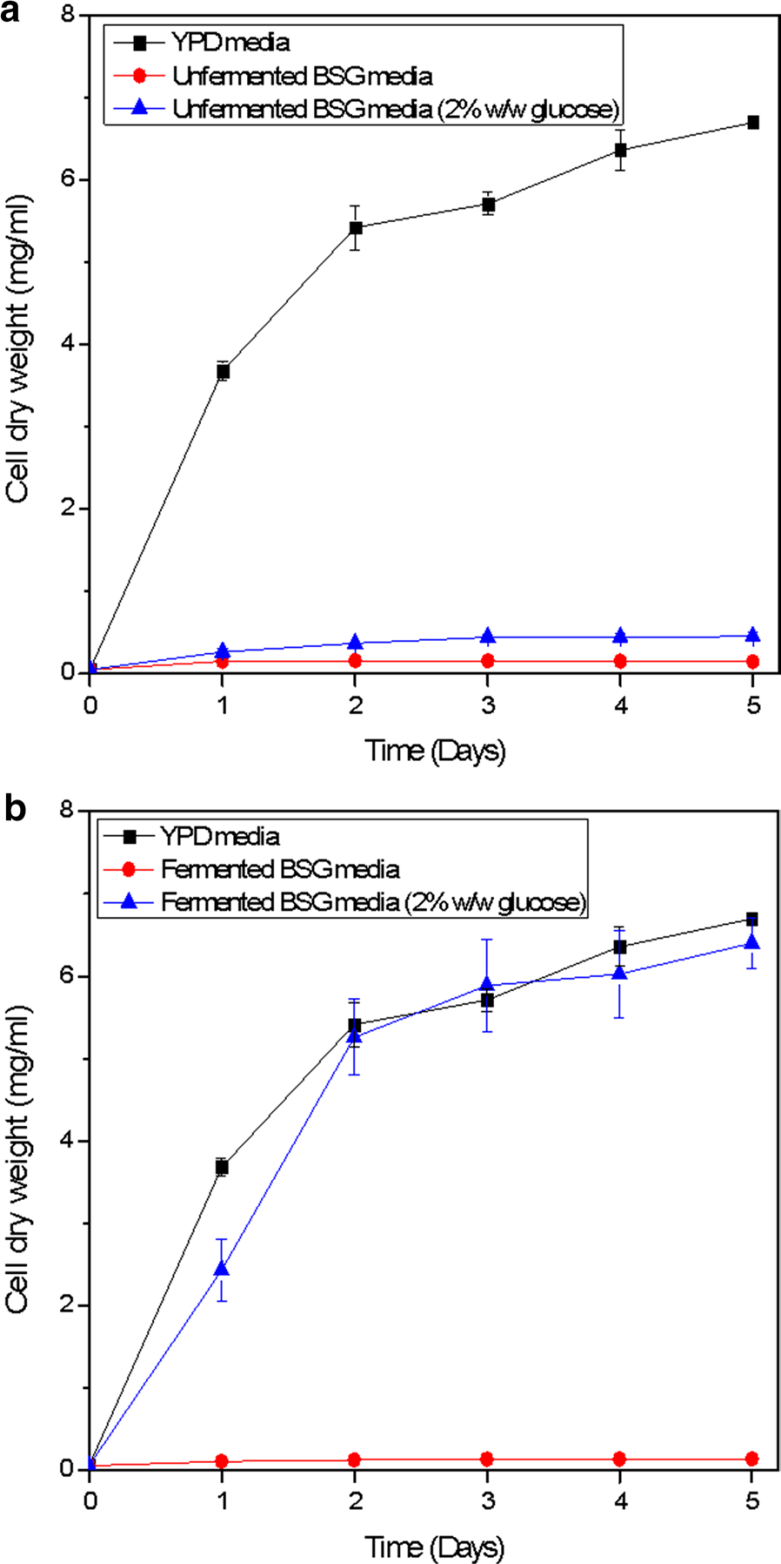
Growth curve for *S. cerevisiae* when grown in **a** unfermented BSG media and **b** fermented BSG media compared to the growth in YPD, represented as cell dry weight (mg/ml) vs. time (days)

Moreover, a similar growth curve was observed in *S. cerevisiae* (Fig. 7) when experimented with the YPD media, fermented BSG (2% w/w glucose) media and unfermented BSG (2% w/w glucose) media.

### Metabolic study of the growth media

The profiling data from the metabolomics analysis demonstrated a complex variation of metabolites in the fermented BSG media compared to the unfermented. This fact was further validated by the clustering analysis. The clustering analysis also demonstrated close similarity in the fermented BSG media and YP media, which partially explained why *R. toruloides* exhibited similar growth patterns in both these media.

According to the significant analysis performed on the identified metabolite profiling data, fermented BSG media reported higher concentrations of essential amino acids (l-threonine, l-valine, phenylalanine, l-leucine, l-lysine), non-essential amino acids (alanine, asparagine, l-cysteine, l-glutamic acid and serine), gluconic acid, butanedioic acid, l-5-oxoproline, l-ornithine, phosphonic acid, putrescine, stearic acid and uracil when compared to unfermented BSG media. According to this data it is clear that the biofermentation has increased the availability of number of essential and non-essential amino acids along with other compounds.

Meanwhile, the fermented BSG media demonstrated similar or higher concentrations in l-valine (essential amino acid), non-essential amino acids (alanine, l-cysteine and l-glutamic acid), 2-butenedioic acid, 2-hydroxyisocaproic acid, gluconic acid, butanedioic acid, L-5-oxoproline, propylene glycol, putrescine, stearic acid and uracil when compared with those identified in YP media.

According to the metabolomics profiling data, it further confirms that the biofermentation step and the autolysis during the nutrient extraction has increased the availability of simpler nitrogenous compounds, especially amino acids, present in the media.

In this study, we aim to extract nutrients present in the BSG and develop a novel growth media for yeasts. The prepared growth media was compared against conventional YPD media. None of the BSG media was able to fulfill the requirement of a carbon source. Fermented BSG media successfully fulfilled the requirement of a nitrogen source required for yeast growth. The biological pretreatment and autolysis enhanced the novel media by improving the accessibility of nitrogen sources. Fatty acid production and carotenoid production of *R. toruloides* in fermented BSG media were reported in comparable levels to those grown in YPD media.

Development of this novel media from BSG will be a more sustainable option while offering a cost-effective alternative for expensive nitrogen sources for yeast cultivation.

## Abbreviations

ANOVA: analysis of variance
BOD: biological oxygen demand
BSG: Brewers’ spent grain
FAME: fatty acid methyl esters
FDR: false discovery rate
GCMS: gas chromatography mass spectrometry
HPLC: high performance liquid chromatography
HSD: honest significant difference
IS: internal standard
PCA: principal component analysis
YPD: yeast extract-peptone (2% w/w glucose)
